# Winging it: hummingbirds alter flying kinematics during molt

**DOI:** 10.1242/bio.060370

**Published:** 2024-11-11

**Authors:** Andrés F. Díaz-Salazar, Felipe Garzón-Agudelo, Ashley Smiley, Carlos Daniel Cadena, Alejandro Rico-Guevara

**Affiliations:** ^1^ Laboratorio de Biología Evolutiva de Vertebrados, Departamento de Ciencias Biológicas, Universidad de Los Andes, Carrera 1 No. 18 A 10, Bogotá 111711, Colombia; ^2^ Colibrí Gorriazul Research Center, Fusagasugá, Cundinamarca 252217, Colombia; ^3^ Department of Integrative Biology, University of California, Berkeley, 3040 Valley Life Sciences Building, Berkeley, CA 94720, USA; ^4^ Department of Biology, University of Washington, Seattle, WA 98195, USA; ^5^ Burke Museum of Natural History and Culture, University of Washington, Seattle, WA 98105, USA

**Keywords:** Hovering, Hummingbirds, Lift, Molt, Flight kinematics, Annual cycle

## Abstract

Hummingbirds are well known for their hovering flight, one of the most energetically expensive modes of locomotion among animals. Molt is a costly event in the annual cycle, in which birds replace their feathers, including all their primary feathers, which, in hummingbirds, comprise most of the area of the wing. Despite this, the effects of molt on hovering flight are not well known. Here, we examined high-speed videos (14 individuals of three species from the Colombian Andes recorded at 1200 frames per second) comparing molting and non-molting hummingbirds’ wing kinematics and wingtip trajectories. We found that molting hummingbirds rotated their wings in more acute angles during both downstroke and upstroke compared to non-molting individuals (10° versus 20°, and 15° versus 29°, respectively), while other flight parameters remained unchanged. Our findings show that hummingbirds are capable of sustaining hovering flight and thereby maintaining their weight support even under impressive wing area reductions by adjusting their stroke amplitudes.

## Teaser

**Summary:** Hummingbirds sustain hovering flight during molt by adjusting wing angles, despite reduced wing area, maintaining weight support as shown in high-speed video analysis of Colombian Andes species.

## INTRODUCTION

Birds experience various developmentally demanding events throughout life, from reproduction to migration, that can be especially limiting periods in the annual cycle (Bridge, 2011). Among these events, molt (i.e. the periodic shedding and replacement of feathers; Palmer, 1972) is relatively understudied in highly specialized flying birds despite its serious implications for flight performance. For instance, birds exhibit increased metabolic rates during molting (Walsberg, 1983) and undergo significant morphological changes throughout the process, such as wing gaps caused by feather shedding (Chai, 1997; Walsberg, 1983). Flight feathers function as airfoils that generate lift (Rayner, 1988). Therefore, the loss of flight feathers may result in kinematic adjustments as well as changes in the resultant aerodynamic forces generated in order to meet the demand of supporting the body weight of volant avian taxa during flight. Yet it is likely that molt does not affect all avian groups in the same manner, given the extreme variation across birds in their body plans (McNab, 1988), metabolic rates (Shankar et al., 2019; Tucker, 1973), and molt strategies (Chai and Dudley, 1999; Langston and Rohwer, 1996; Palmer, 1972; Swaddle and Witter, 1997).

Hummingbirds, in particular, face significant challenges during molt periods. They have the highest mass-specific metabolic rates among vertebrates, in part due to the extreme energetic demands of their hovering flight (Altshuler and Dudley, 2002; Suarez, 1992), and produce lift during both the downstroke and upstroke of wing-flapping (Hedrick et al., 2012; Tobalske et al., 2007; Warrick et al., 2005; Weis-Fogh, 1972), following an ‘insect-like’ flying pattern that is dissimilar to other birds (Cheng et al., 2016). This pattern is characterized by a figure-eight wingtip trajectory during steady hovering (defined as maintaining a forward flight speed equal to zero; Magnan and Sainte-Laguë, 1933), which is lost during fast forward flight when the direction of the aerodynamic forces is modified (Tobalske et al., 2007; Cheng et al., 2016). Hummingbirds’ skeletomuscular structure and complex nervous system allow them to control their wingtip trajectories, body position, and ultimately their flight kinematics, enabling rapid responses to external stimuli (e.g. during escape maneuvers; Cheng et al., 2016). During static hovering, molting and non-molting hummingbirds alike must produce enough lift force to support their body weight. If we assume no changes in body weight, a reduction in the airfoil area during molt, an energetically stressful event (Chai, 1997), is likely to affect the kinematics of a wingbeat cycle (Achache et al., 2017; 2018), potentially resulting in changes in the aerodynamic forces generated to compensate for the loss of wing feathers.

We currently lack a comprehensive understanding of how molt impacts hummingbird hovering kinematics. Previous research by Chai in 1997 on the Ruby-throated Hummingbird (*Archilochus colubris*), found no significant effects of molt on aerodynamic variables associated with hovering, such as stroke amplitude, and a small but significant reduction in wing frequency. However, this laboratory study was focused solely on analyzing birds’ hovering sequences from one lateral perspective, which were video recorded using a mirror oriented above the bird at a 45° angle to the horizontal. Chai noted that, in future work, high-speed video and vortex visualization techniques would be imperative to precisely determine any molt-related effects on flight performance. Furthermore, a recent study by Achache et al. (2018) suggested that, during molting, hummingbird wings may exhibit diminished capacity to generate lift during the downstroke, as indicated by vortex and force measurements. Notably, these analyses were conducted on wing models rather than on live, free-flying birds. Therefore, the influence of molt beyond captive or otherwise manipulated settings, especially from multiple perspectives of flight projections (i.e. different camera angles), remains unexplored. Given that captivity is associated with altered growth, feather quality (Butler et al., 2010), and behavior in birds, including hummingbirds (Zenzal et al., 2014), a comprehensive understanding of how molt affects hummingbird hovering kinematics necessitates further research, particularly employing techniques that capture the dynamics of free-ranging birds in natural environments.

Hummingbirds demonstrate exceptional flight capabilities, sustaining flight demands consistently throughout their annual cycle, even when experiencing up to a 40% reduction in total airfoil planform area (the wing area visible from above) during molt (Fig. S1B). A bird's weight during flight is supported in proportion to its airfoil planform area (Feinsinger and Chaplin, 1975). Following a reduction in its planform area (e.g. due to molting), a hummingbird must generate the same time-averaged lift force (equal to its body weight) that it would produce with intact wings in order to engage in steady hovering flight. Possible mechanistic techniques to compensate for the loss of flight feathers, while still meeting the demands of hovering flight, include modifying flight kinematics (Achache et al., 2018; Cheng et al., 2016) and/or mass loss (Chai, 1997). Focusing on flight kinematics, we predict that molting hummingbirds will alter their flight parameters to overcome the morphological changes owed to molt. Specifically, we expect to observe an increased wingspan stroke amplitude in molting birds relative to non-molting birds, as higher stroke amplitude angles accelerate a larger mass of air (Altshuler and Dudley, 2003), or hummingbirds can sustain stroke amplitude but increase flapping frequency to achieve equivalent average forces (Altshuler et al., 2005; Ellington, 1984). We also expect changes in the figure-eight wingtips’ trajectories and body positional angles that would reflect re-orientation of the aerodynamic forces (Cheng et al., 2016; Fernández et al., 2012).

Other kinematic variables that could be modified during molting events are the longitudinal axis wing rotation, and angle of attack. Two lengthwise rotations of a hovering wing occur during supination and pronation in order to achieve sufficient angles of attack that generate enough lift force to offset body weight (Vogel, 1981). As lift coefficients for a given airfoil increase non-linearly with angle of attack (Vogel, 1981), fine-tuning the timing of the two rotations may allow for increased angles of attack and resultant peak lift force generation to occur during parts of the wingbeat cycle (Sane and Dickinson, 2002) that could help compensate for a reduction in planform area. However, the orientation of our video data does not allow us to take measurements on the timing of the longitudinal axis rotation events of the wings. In addition, we were unable to measure the angle of attack due to the lighting conditions of our videos. As a whole, the time-averaged lift force production remains constant and equal to the hummingbird's body weight.

**Fig. 1.**
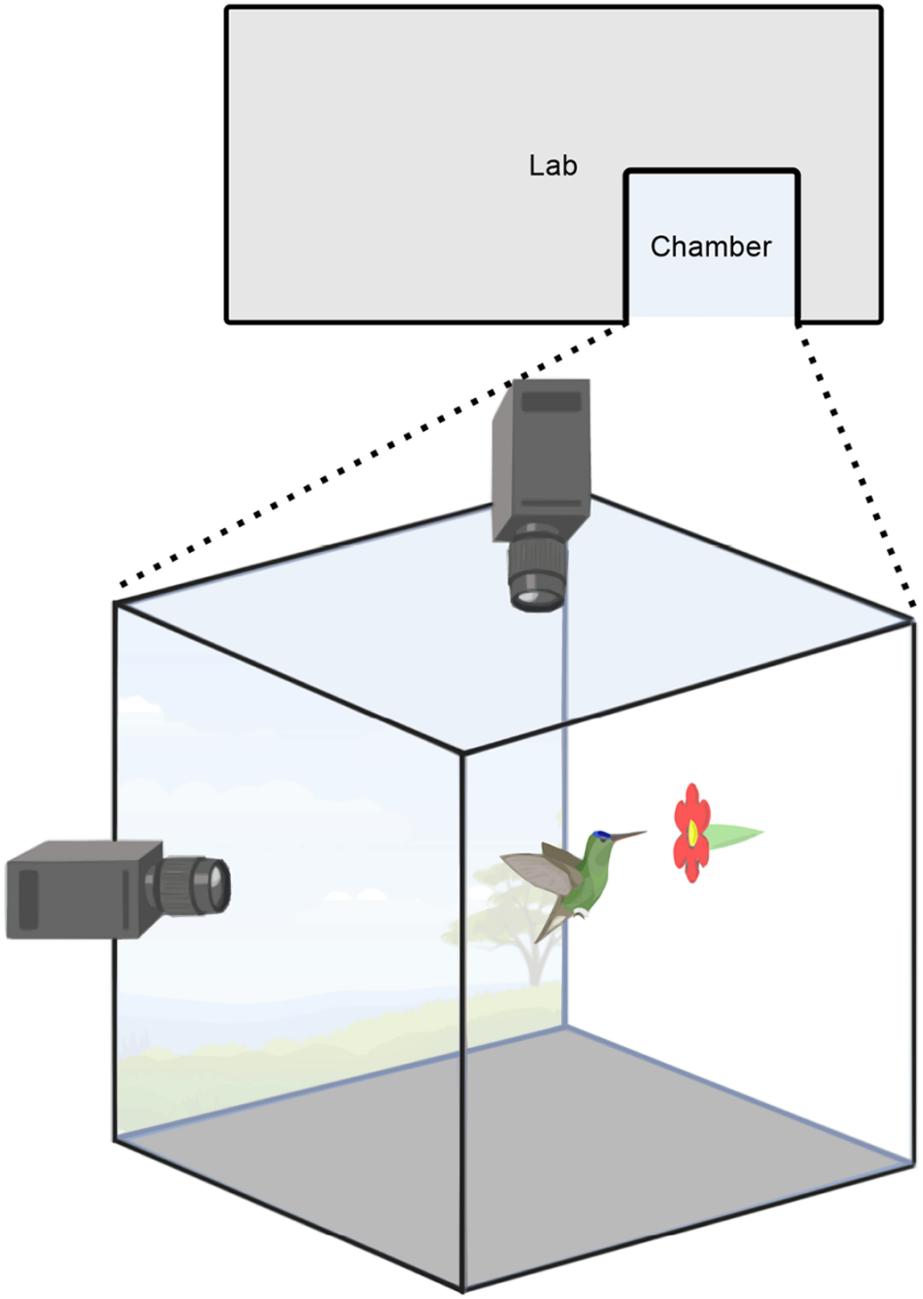
**Experimental setup for high-speed video recording of hummingbirds during static hovering.** A chamber connected to an open laboratory window allowed free-ranging hummingbirds access to an artificial flower which provided a 20% sucrose solution. High-speed cameras positioned at 90° angles simultaneously captured top and side views of the hummingbirds’ hovering flight.

**Fig. 2.**
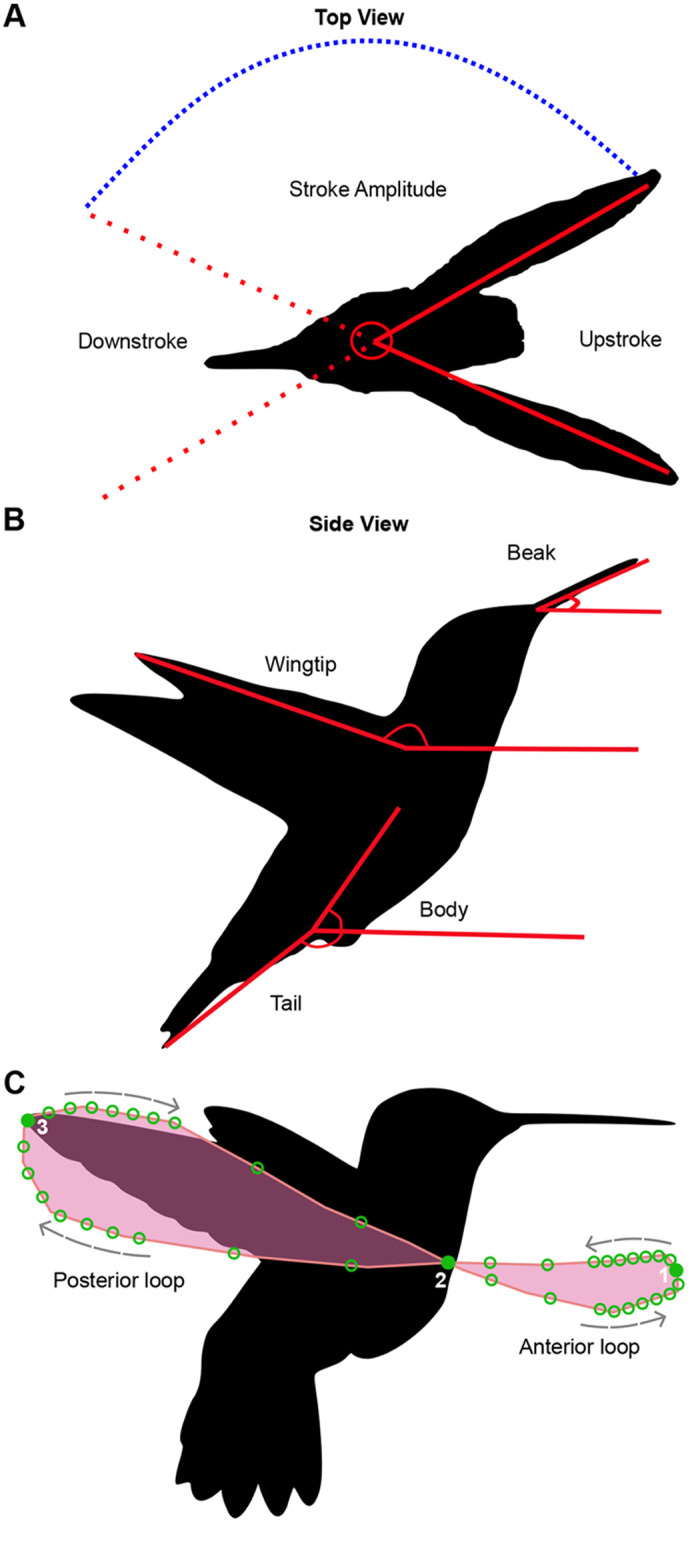
**Kinematic variables located over the top view and side view.** (A) Representation of the top view at maximum positional angle for the upstroke (solid red line) and minimum positional angle for the downstroke (red dotted line), defined as the angles formed between the wingtips with respect to the posterior-most point of the head, at the end of upstroke and downstroke. The stroke amplitude was defined as the angle between the positions of the tip of one wing at the end of upstroke and downstroke with respect to the posterior-most point of the head. (B) Body positional angles considered for the side view perspective. (C) Analemma (i.e. figure-eight pattern) formed by the wingtip trajectory during a complete wingbeat (pink line) and landmarks (numbered filled circles) and semilandmarks (open circles) that characterize it. Arrows represent the direction of wingtip movement.

In this study, we evaluate the effect of molt on the hovering kinematics and flying patterns of three species of hummingbirds in the Colombian Andes, using high-speed video and geometric morphometric analysis (Figs 1, 2). To our knowledge, this is the first empirical study on the flight kinematics of free-ranging hummingbirds during molt.

## RESULTS

### Hovering kinematics

**Fig. 3.**
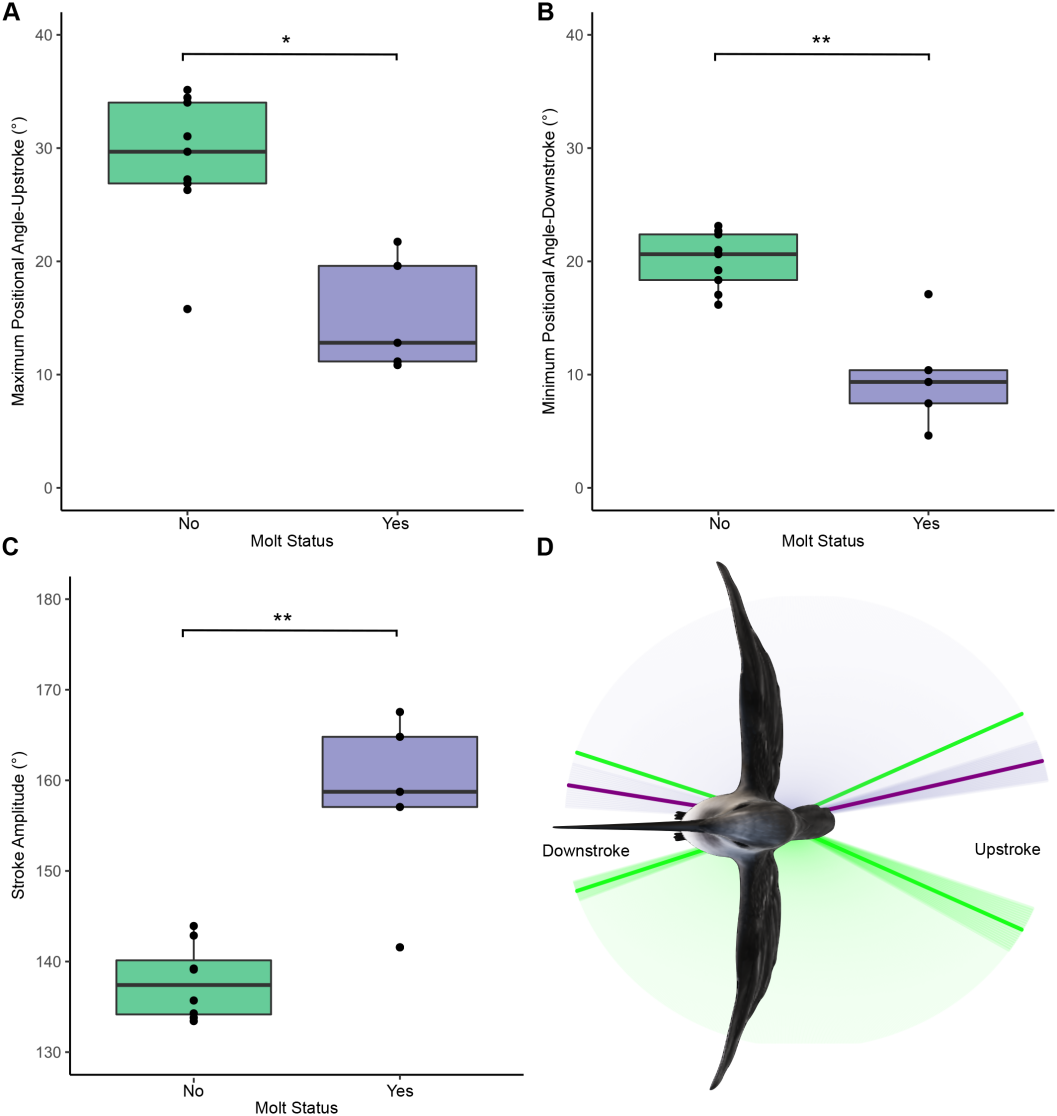
**Top-view measurements revealing that molting hummingbirds showed higher stroke amplitudes.** (A) Wing maximum positional angles (upstroke) (B) and minimum positional angle (downstroke) were more acute in molting hummingbirds, and (C) stroke amplitudes were higher. This suggests molting hummingbirds moved their wings closer to each other in each wing beat during upstroke and downstroke. As a result, (D) molting hummingbirds (right wing in purple) showed larger wingtip trajectories than non-molting hummingbirds (left wing in green). Solid lines showing stroke amplitude angle average and shades represent their standard deviation. The non-molting stroke average is also shown in green on the right wing to facilitate comparison. Purple represents molting and green non-molting.

Based on the top-view video analysis, molting and non-molting hummingbirds differed in stroke amplitude, minimum positional angles (downstroke), and maximum positional angles (upstroke) (average change of 157° versus 138°, *P*<0.001; 10° versus 20°, *P*<0.001; and 15° versus 29°, *P*<0.004, respectively; Fig. 3). Molting hummingbirds showed more acute terminal angles during their downstroke and upstroke, resulting in higher stroke amplitudes compared to non-molting hummingbirds (Fig. 3). Our results suggest that molt was the only covariate in our models that significantly affected flight kinematics. Species and the interaction between species and molt did not affect them (Table S1). In contrast, in the side view angles (beak, tail, body, and wingtips), we did not find any significant differences in positional angles between molting and non-molting birds (*P*>0.05 in all cases; Fig. S2). Additionally, molting birds exhibited similar wing-flapping frequencies compared to non-molting birds (*P*=0.267; Fig. S2).

### Wingbeat patterns: analemma size and shape

**Fig. 4.**
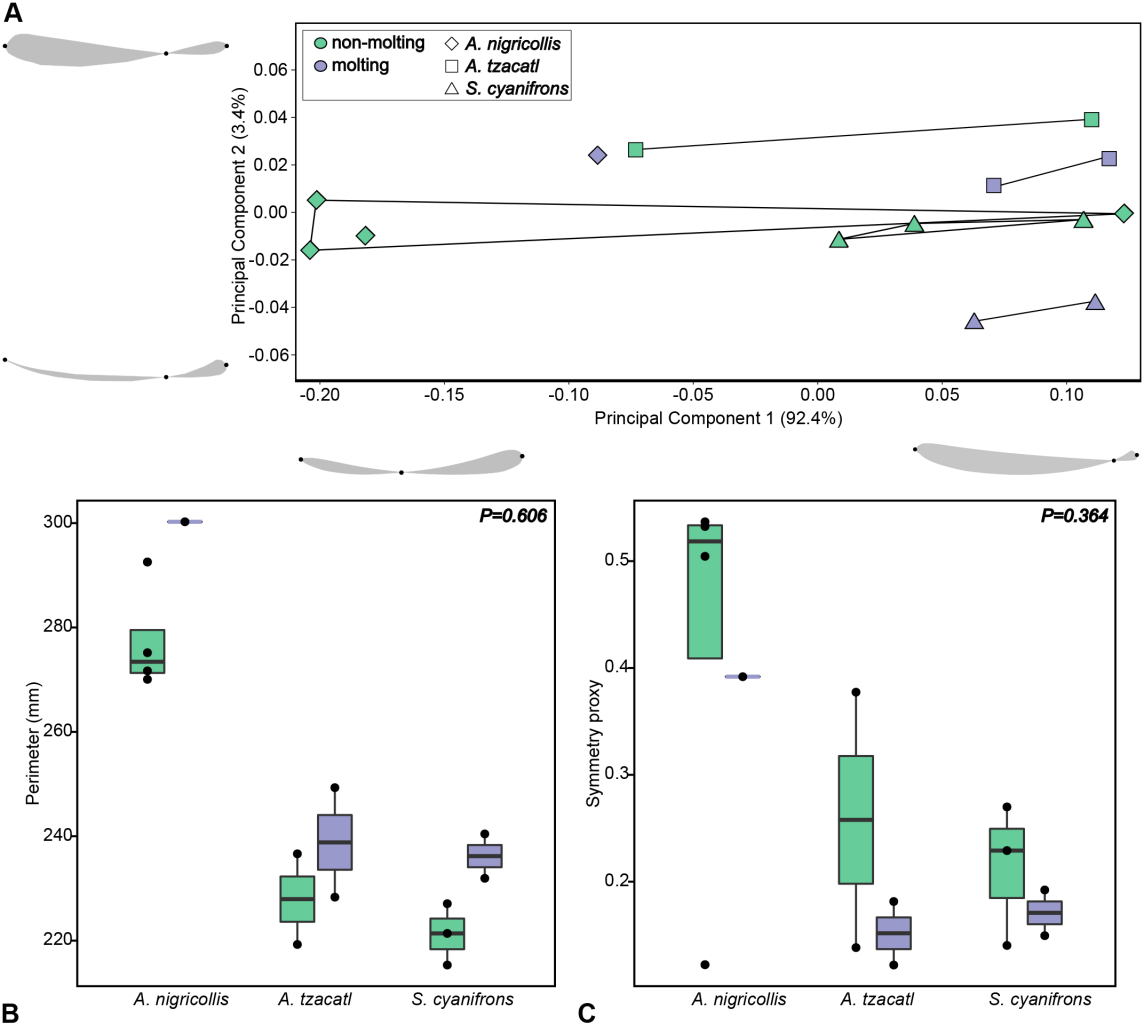
**Molting status did not affect analemma size or shape.** (A) First 2 principal components representing analemma shape space. Wireframes represent the shape variation associated with the extremes of the PCs. Semilandmarks are not shown. Wireframes have the same orientation as Fig. 2C. (B) Analemma perimeter and (C) ratio of anterior loop to analemma length for molting and non-molting individuals per species. Statistical differences were evaluated between all molting and non-molting individuals (*P* values in B and C). Purple represents molting and green non-molting individuals.

We observed no significant differences between molting and non-molting birds in the perimeter of the analemmas or in the ratio of the anterior loop to the length of the analemma (*P*=0.606, *P*=0.364, respectively; Fig. 4). Additionally, we did not find any significant allometric effect (R^2^=0.19, *P*=0.117).

The principal component analysis (PCA) revealed that the first two principal components combined accounted for 95.83% of the total variation in the shape of the analemmas (PC1=92.44%, PC2=3.39%; Fig. 4). The Procrustes ANOVA indicated that the symmetry proxy explained the majority of analemma shape variation (R^2^=0.92, *P*=0.001), representing a simple alternative to characterize analemma shape. PC1 showed a strong association with this metric. Specifically, analemmas were more symmetric toward the negative side of PC1, corresponding to higher ratio values, and had relatively shorter anterior loops toward the positive side of PC1, corresponding to lower ratio values. PC2 described the proximity of the downstroke and upstroke trajectories, particularly in the posterior loop of the analemmas. There was no clear separation in the analemma shape space between molting and non-molting hummingbirds, as indicated by the Procrustes ANOVA (R^2^=0.11, *P*=0.203; Fig. 4).

Hummingbirds with intact wing area exhibited more variance in analemma shape than molting birds, but the difference in variances was not significant (Procrustes variance was 0.0176 in non-molting birds and 0.0073 in molting birds, *P*=0.118). The same pattern of variation can be visualized per species along PC1 and in the boxplot of the ratio of the anterior loop to the analemma length (Fig. 4).

## DISCUSSION

Here, we examined how molt affects the hovering kinematics, flying patterns and the wingbeat pattern of free-ranging hummingbirds. Our findings suggest that hummingbirds may overcome flight limitations imposed by molt through adjustments in wing angles during both the upstroke and downstroke. Hummingbird wings trace an analemma-like shape during wingbeats but, remarkably, these trajectories remained similar in molting and non-molting hummingbirds. Our results are based on 14 individuals of free-ranging hummingbirds (molting individuals, *n*=5; non-molting individuals, *n*=9), and although our analyses show clear trends in hummingbird flight kinematics, we recognize that a larger sample size is needed for more robust conclusions.

We found that molting hummingbirds exhibit greater stroke amplitude, and more acute upstroke and downstroke terminal angles than non-molting hummingbirds when seen from above (Fig. 3). In other words, molting individuals carry their wings a longer distance, and therefore accelerate a larger mass of air, to complete a single flap than non-molting individuals, in both the downstroke and upstroke. Based on the Rankine-Froude propeller model (Osborne, 1951) if we interpret stroke amplitude as an actuator disk, larger actuator disk areas would ostensibly result in a reduction of induced velocity according to equation 3.7 from Dudley, 2002 [Vinduced=((mg -Lb)/2rA0(V2+Vi2))1/2]. A reduction in induced velocity would thereby lead to a resulting reduction in induced power. Induced power reflects the costs of lift production and equals the product of body weight and induced velocity (Dudley, 2002). Taken together, our findings suggest that molting hummingbirds may be modulating their stroke amplitude in order to support their body weight in hovering flight without accruing a costly increase in induced power. It is important to note that our assumptions are based on theoretical models and that no empirical flow visualizations were measured. Greater stroke amplitudes may compensate for the loss of flight feathers (Chai and Millard, 1997), thus alleviating the morphological limitations imposed by molt.

Conversely, apart from the changes in stroke amplitude observed from a top view, molting hummingbirds did not alter key kinematic and body positional variables relative to non-molting hummingbirds when observed from the side. Despite experiencing substantial reductions in airfoil area, molting individuals managed to maintain consistent positional angles of their wings, bill, body, and tail. Additionally, during hovering, molting hummingbirds maintained their wingtip trajectory (wingbeat pattern), as shown in our geometric morphometric analysis. We expected to observe alterations in the figure-eight trajectory (analemma) traced by hummingbirds’ wingtips between molting and non-molting hummingbirds, which would reflect a compensatory mechanism against molt. However, we found no differences between molting and non-molting birds in the shape or perimeter of their analemmas. Although the molting state did not have a significant effect on the perimeter of the analemma, in each species, molting birds consistently exhibited higher maximum perimeter values compared to non-molting birds (Fig. 4B). This finding aligns with the increased stroke amplitudes observed in our kinematic analysis.

Wing area reductions generally result in greater wing-loading, which could be expected to lead to behavioral and locomotor compensations (Swaddle and Witter, 1997). For instance, birds could reduce their body mass to alleviate kinematic constraints (Achache et al., 2018; Chai, 1997). However, it remains unclear whether hummingbirds experience weight loss during molting, whether this occurs across species and sexes, and the extent to which body mass modulation can be achieved in the wild. Furthermore, it is uncertain whether hummingbirds can voluntarily control their body mass to compensate for the reduction in wing area while maintaining their ability to generate lift, since more weight and less airfoil area would imply greater wing loading (Chai, 1997; Swaddle and Witter, 1997), or whether, given the metabolic demands of molting (Walsberg, 1983), changes in mass instead reflect increased physiological demands of feather synthesis (Swaddle and Witter, 1997). Because we did not measure body mass, we were unable to determine whether the species we studied reduced their mass during molt, as has been seen in other birds (e.g. penguins, Cherel et al., 1994; and crows, Lind and Jakobsson, 2001).

Another way to compensate for potentially detrimental effects of molt on hovering may be to increase wing flapping frequency, thereby reaching equivalent aerodynamic forces to those produced via high-amplitude flapping (Altshuler et al., 2005; Fernández et al., 2012; Rajabi et al., 2020). However, we found no differences in flapping frequency between molting and non-molting hummingbirds. In contrast, molting birds traced larger wing trajectories, maintaining on average the same flapping frequencies compared to non-molting birds, which would imply higher flapping velocities to achieve larger wing trajectories.

Our study, constrained by the available sample sizes, did not extensively explore interspecific or intraspecific variation of strategies to cope with aerodynamic restrictions related to molt (Wilcox and Clark, 2022). The results of our models did not suggest a significant effect of species on the variables we assessed as proxies for flight kinematics (Table S1). However, we recognize that hummingbirds, which are characterized by diverse ecological strategies (e.g. migration and foraging strategies; Sargent et al., 2021) and evolutionary histories (McGuire et al., 2014), exhibit significant variation in wing morphology and flight physiology (Altshuler and Dudley, 2002), and it is likely that different compensatory mechanisms exist to overcome molt effects in flight kinematics across the wide diversity of hummingbird species. Consequently, compensatory mechanisms reducing the impact of molt may vary among different clades, as suggested by our GLM that compared top view variables between clades (Table S3, Fig. S2A). Recognizing the importance of robust group estimates, including means and variances, to reach meaningful conclusions (Cardini et al., 2015), we encourage the use of larger sample sizes to perform intra- and interspecific analyses. We recommend the future use of three-dimensional analysis to avoid the potential loss of information caused by the two-dimensional projection of a three-dimensional motion pattern (Cardini, 2014). Furthermore, our analysis did not assess the individual effects of single feather gaps. During molt, birds are likely to experience different effects on flying performance and energetic expenditure, depending on which feathers are shedding or being replaced. For a rotating, solid body about an axis, the tangential velocity of a point along the body is proportional to its distance from the axis of rotation (Vogel, 1981). If we treat the hummingbird's shoulder as an axis of rotation, the tangential velocity of each feather is proportional to its distance from the shoulder. Therefore, the distal primary feathers are theoretically moving faster than the proximal secondary feathers. Lift for airfoils is approximately proportional to the second power of tangential velocity (Vogel, 1981), and thus, the loss of primary feathers would incur more need for kinematic compensation versus the loss of secondary feathers. Although there is a lack of information regarding how molt influences the life history of hummingbirds, the molt pattern is notably reversed for primaries IX and X in hummingbirds, possibly as an adaptation to mitigate the effects of molt on lift performance (Baltosser, 1995; Chai, 1997; Stiles, 1995). Our analysis focused solely on hovering flight, thus excluding considerations for forward flight, acceleration, and maneuverability. Nonetheless, it is remarkable to witness the exceptional flying abilities of these avian species, which consistently surpass physical and physiological constraints even during molting periods (Graham et al., 2016). We encourage future research endeavors to explore the effects of molt on more intricate flying maneuvers, such as rolls, pitches, and yawing, and to approach molt-related impacts from diverse perspectives encompassing physiology, behavior, and ecology.

## MATERIALS AND METHODS

### Data collection

We conducted all of our work at the Colibrí Gorriazul Research Station, located on the western slope of the Eastern Cordillera of the Colombian Andes (04°23′N 74°21′W), at an elevation of 1700 m. Birds were captured and sampled using either mist nets or drop-net feeder traps (Russell et al., 2001). Prior to recording, we tagged hummingbirds with 7-mm passive integrated transponders (PIT tags), which we implanted subcutaneously between the scapulae (Hou et al., 2015); to allow for full wing motion during backward rotation, we placed these tags between the thoracic and lumbar vertebrae, avoiding any obstruction (Bandivadekar et al., 2018). We used these PIT tags as an individual identification technique in lieu of tarsal banding, releasing each bird and monitoring its return to a station feeder equipped with a radio-frequency identification (RFID) antenna.

Next, we used two FASTEC^®^ IL5 (Fastec Imaging, San Diego, CA, USA) high-speed cameras to record both molting and non-molting hummingbirds within a semi-controlled environment: a transparent Plexiglas chamber (90 cm×90 cm×90 cm). The recessed chamber was connected to a laboratory window that opened to the outside (Fig. 1), where feeders were strategically positioned to attract free-ranging hummingbirds. Inside the chamber, a 10 ml syringe was connected to an artificial flower, which provided a 20% sucrose solution *ad libitum*. Cameras were positioned 90° from each other, enabling simultaneous recording of both top and side views of the artificial flower and the visiting hummingbirds.

Hummingbirds voluntarily entered the chamber to access the artificial nectar provided through the syringe. Following an acclimation period (approximately 15 days), we recorded high-speed videos at 1200 frames s^−1^ for individuals of three species of resident hummingbirds (Indigo-capped Hummingbird, *Saucerottia cyanifrons;* Rufous-tailed Hummingbird, *Amazilia tzacatl;* and Black-throated Mango *Anthracothorax nigricollis*). We filmed 14 sets of synchronized top and side view videos for each individual: five sets for molting birds and nine sets for non-molting birds (see detailed sample size in Table S2). The RFID antenna readings confirmed that each of the recorded birds was a different individual. For our analysis, we focused exclusively on measuring static hovering flight between drinking bouts. No measurements were taken while individuals were actively engaged in nectar consumption, rotating, or moving across different planes (excluding flying sequences wherein we detected horizontal or vertical movement of the individual in any of the views). We did not systematically assess specific molt stages, but simply recorded the presence or absence of wing molt. The total number of hummingbird individuals limits the robustness of statistical inferences for hypothesis testing; however, we qualitatively split our data into four molting categories and present these results in the Supplementary Information (Figs S1, S2). Furthermore, because our study emphasized free-flying individuals that went in and out from our study set, we were not able to measure their weight, and so we did not account for potential effects of body mass, which varies between the studied species (mean body mass: 5 g for *S. cyanifrons* and *A. tzacatl,* and 7 g for *A. nigricollis;* Dunning, 2007).

### Hovering kinematics

To assess the impact of molt on hovering kinematics, we chose consistent body landmarks from individuals to look for alterations in their positional angles (Fig. 2A). First, we converted each video to a stack of images to conduct a frame-by-frame analysis. We analyzed five complete wingbeat cycles (i.e. one downstroke and one upstroke per cycle) for each set of synchronized videos. We used the software Fiji (ImageJ; Schindelin et al., 2012) to measure flapping frequency, stroke amplitude, and angles describing the position of selected body parts as indicators of flight kinematics (Fig. 2A,B) following previous studies (Achache et al., 2018; Altshuler et al., 2005; Chai, 1997). We measured the variables separately for each wingbeat cycle's downstroke and upstroke.

For the top-view kinematics, we quantified maximum and minimum wingtip positional angles for downstroke and upstroke, respectively. To do this, we traced the angles between both wingtips and the posterior-most point of the head as the vertex at the exact point where the wings reached their maximum point before the start of the next flapping cycle (Fig. 2A). For the side view, we measured the positional angles of four body parts by digitizing points on the beak (base and tip of the beak), tail (base and tip of the tail), body (base of the tail and mid-body point), and wingtips (shoulder and wingtip) (Fig. 2B). Additionally, following Groom et al. (2018), we estimated flapping frequency by dividing the recording frame rate (1200 frames s^−1^) by the total number of frames that were necessary for a hummingbird to complete a single flap. We then took the average from five consecutive flap cycles in each video.

### Wingbeat patterns: analemma size and shape

To assess whether molting hummingbirds exhibited altered hovering patterns compared to non-molting hummingbirds, we analyzed the lemniscate-shaped stroke cycle (figure-eight pattern) traced by wingtips during each wingbeat, hereafter referred to as an analemma (Fig. 2C). Using a two-dimensional geometric morphometric approach, we assessed differences in size and shape of analemmas between molting and non-molting hummingbirds from the side-view video sets. We limited our analysis of wingtip trajectories to the lateral projection because we expected most of the shape variation to be concentrated in this plane (Tobalske et al., 2007). More specifically, we expected the variation in the wingtip trajectory in the dorsal projection to be small, because it is constrained by the kinematic rigidity of hummingbird wings (i.e. low degree of wing flexion during flapping), which is especially higher during static flight (Tobalske et al., 2007). For each video, we traced the wingtip trajectory over a minimum of three complete wingbeat cycles, using the plugin MtrackJ (Meijering et al., 2012) in the Fiji software (Schindelin et al., 2012). After digitizing the videos, we obtained a total of 85 analemmas, 30 for molting birds and 55 for non-molting birds (Table S2).

To analyze the shape and size of the analemmas, we digitized three landmarks and 36 semilandmarks using TpsDig2 (Rohlf, 2021a; Fig. 2C) through the following process: (1) three landmarks were digitized (1-anterior-most point of the analemma; 2- point of intersection between upstroke and downstroke trajectories; 3- posterior-most point of the analemma; Fig. 2C); (2) 20 equally spaced points were digitized along each curve, giving a total of 80 semilandmarks and 3 landmarks; (3) We estimated how many landmarks could be removed without sacrificing shape characterization using the LaSEC function in the LaMBDA R package (Watanabe, 2018) (Fig. S3); (4) 11 points were removed from the straight portions of each curve, giving the final number of semilandmarks (36) and landmarks (3) used. Digitizing semilandmarks in this way allowed them to concentrate in the regions with more shape variation (extremes of the analemmas) and remove non-informative points.

We calculated the perimeter of each analemma by summing distances between landmarks and semilandmarks using the *interlmkdist* function in the *geomorph* package for R (Baken et al., 2021). Because the perimeter and centroid size were strongly correlated (r=0.99, *P*=0.0001), we focused solely on the perimeter as a size variable for subsequent analysis. As a proxy for symmetry, we defined a length proportion corresponding to each analemma. We described its shape as consisting of two loops, one anterior and one posterior (Fig. 2C). We then calculated the ratio between the length of the anterior loop (distance between landmarks 1 and 2) and the overall length of the analemma (distance between landmarks 1 and 3; Fig. 2C).

We obtained the shape variables (Procrustes tangent coordinates) through a Generalized Procrustes analysis (GPA; Rohlf, 1999) using the function *gpagen* from *geomorph*. This analysis effectively accounted for non-shape variation stemming from differences in position, scale, and orientation. For the GPA procedure, semilandmarks were allowed to slide under the criterion of bending energy minimization (Gunz and Mitteroecker, 2013). We decided not to use Procrustes distance minimization for semilandmark sliding because this method produced highly distorted configurations on our dataset. Some semilandmarks slid past neighboring landmarks and semilandmarks, changing their relative positions. This is probably due to the large shape variation in our sample (Gunz and Mitteroecker, 2013), in which some configurations differ greatly from the average shape, meaning that semilandmarks must slide greater distances to minimize shape differences. The resulting Procrustes coordinates were orthogonally projected from a curved space to a flat tangent space, yielding Procrustes tangent coordinates (Rohlf, 1999; Mitteroecker and Gunz, 2009). To determine if the flat tangent space is a good approximation of the curved shape space, a correlation of the Euclidean and Procrustes distances between all pairs of individuals was performed using TpsSmall (Rohlf, 2021b). The correlation was high (r= 0.999801) indicating that shape differences between individuals are not distorted by the projection to the tangent space.

Subsequently, we averaged perimeter and Procrustes tangent coordinates by individual. To obtain a representation of shape space, we performed a Principal Component Analysis (PCA) using the covariance matrix of the Procrustes tangent coordinates (*gm.prcomp* function from *geomorph*). We then generated wireframes to visually depict shape variation associated with the extremes of the PCA axes (Klingenberg and Rohwer, 2013).

### Statistical analyses

To statistically assess differences between molting and non-molting kinematics we conducted generalized linear models (GLMs) with the *glm* function from the stats package (R Core Team, 2022). We conducted a separate GLM per variable and considered molt state (presence/absence of molt), species, and the interaction between molt and species, as fixed effects to test for any influence on hovering kinematics.

We assessed the effect of molt on the analemma perimeter and the ratio of anterior loop to analemma length (symmetry proxy) with Mann–Whitney U tests. For analemma shape, we performed Procrustes ANOVA with permutation procedures (Goodall, 1991), wherein the Procrustes tangent coordinates (which correspond to shape) were the response variable and molting state was the categorical variable (using the *procD.lm* function from *geomorph*). We also performed additional Procrustes ANOVA to assess allometry (changes in analemma shape related to changes in analemma perimeter) and the relation between analemma shape and our proportional metric of symmetry (i.e. the ratio of anterior loop to analemma length). To quantify and compare the variation in analemma shape (Procrustes variance) between molting and non-molting birds (Zelditch et al., 2012), we used the *morphol.disparity* function from *geomorph*. All analyses were conducted in R version 4.2.1 (R Core Team, 2022; http://www.R-project.org/).

We assessed the influence of species on each kinematic and morphometric variable by including the species effect as a covariate in our models. We also considered clade effects by running separate models (Table S3), as our species belong to different evolutionary clades (Mangos: *A.nigricollis*; Emeralds: *S. cyanifrons* and *A. tzacalt*; McGuire et al., 2014). Due to our sample size, we do not present inter and intraspecific statistical testing in the Results section. Statistical inferences regarding clade effects on flight kinematics can be found in the Supplementary Information.

## Supplementary Material

10.1242/biolopen.060370_sup1 Supplementary information

## References

[BIO060370C1] Achache, Y., Sapir, N. and Elimelech, Y. (2017). Hovering hummingbird wing aerodynamics during the annual cycle. I. complete wing. *R. Soc. Open Sci.* 4, 170183. 10.1098/rsos.170183 28878971 PMC5579086

[BIO060370C2] Achache, Y., Sapir, N. and Elimelech, Y. (2018). Hovering hummingbird wing aerodynamics during the annual cycle. II. Implications of wing feather molt. *R. Soc. Open Sci.* 5, 1-8. 10.1098/rsos.171766 PMC5830773 29515884

[BIO060370C3] Altshuler, D. L. and Dudley, R. (2003). The ecological and evolutionary interface of hummingbird flight physiology. *J. Exp. Biol.* 205, 2325-2336. 10.1242/jeb.205.16.2325 12124359

[BIO060370C4] Altshuler, D. L., Dickson, W. B., Vance, J. T., Roberts, S. P. and Dickinson, M. H. (2005). Short-amplitude high-frequency wing strokes determine the aerodynamics of honeybee flight. *Proc. Natl. Acad. Sci. USA* 102, 18213-18218. 10.1073/pnas.0506590102 16330767 PMC1312389

[BIO060370C5] Baken, E. K., Collyer, M. L., Kaliontzopoulou, A. and Adams, D. C. (2021). geomorph v4.0 and gmShiny: enhanced analytics and a new graphical interface for a comprehensive morphometric experience. *Methods Ecol. Evol.* 12, 2355-2363. 10.1111/2041-210X.13723

[BIO060370C6] Baltosser, W. H. (1995). Annual molt in Ruby-throated and Black-chinned hummingbirds. *Condor* 97, 484-491. 10.2307/1369034

[BIO060370C7] Bandivadekar, R. R., Pandit, P. S., Sollmann, R., Thomas, M. J., Logan, S. M., Brown, J. C., Klimley, A. P. and Tell, L. A. (2018). Use of RFID technology to characterize feeder visitations and contact network of hummingbirds in urban habitats. *PLoS One* 13, e0208057. 10.1371/journal.pone.0208057 30540787 PMC6291107

[BIO060370C8] Bridge, E. S. (2011). Mind the gaps: what's missing of feather molt. *Condor* 113, 1-4. 10.1525/cond.2011.100228

[BIO060370C9] Butler, M. W., Leppert, L. L. and Dufty, A. M., Jr. (2010). Effects of small increases in corticosterone levels on morphology, immune function, and feather development. *Physiol. Biochem. Zool.* 83, 78-86. 10.1086/648483 19929638

[BIO060370C10] Cardini, A. (2014). Missing the third dimension in geometric morphometrics: how to assess if 2D images really are a good proxy for 3D structures? *Hystrix* 25, 73-81.

[BIO060370C11] Cardini, A., Seetah, K. and Barker, G. (2015). How many specimens do I need? Sampling error in geometric morphometrics: testing the sensitivity of means and variances in simple randomized selection experiments. *Zoomorphology* 134, 149-163. 10.1007/s00435-015-0253-z

[BIO060370C12] Chai, P. (1997). Hummingbird hovering energetics during moult of primary flight feathers. *J. Exp. Biol.* 200, 1527-1536. 10.1242/jeb.200.10.1527 9192500

[BIO060370C13] Chai, P. and Dudley, R. (1999). Maximum flight performance of hummingbirds: capacities, constraints, and trade-offs. *Am. Nat.* 153, 398-411. 10.1086/303179

[BIO060370C14] Chai, P. and Millard, D. (1997). Flight and size constraints: hovering performance of large hummingbirds under maximal loading. *J. Exp. Biol.* 200, 2757-2763. 10.1242/jeb.200.21.2757 9418032

[BIO060370C15] Cheng, B., Tobalske, B. W., Powers, D. R., Hedrick, T. L., Wethington, S. M., Chiu, G. T.-C. and Deng, X. (2016). Flight mechanics and control of escape manoeuvres in hummingbirds I. Flight kinematics. *J. Exp. Biol.* 219, 3518-3531. 10.1242/jeb.137539 27595850

[BIO060370C16] Cherel, Y., Charrassin, J. B. and Challet, E. (1994). Energy and protein requirements for molt in the king penguin Aptenodytes patagonicus. *Am. J. Physiol. Regul. Integr. Comp. Physiol.* 266, R1182-R1188. 10.1152/ajpregu.1994.266.4.R1182 8184961

[BIO060370C17] Dudley, R. (2002). *The Biomechanics of Insect Flight: Form, Function, Evolution*. Princeton University Press.

[BIO060370C18] Dunning, J. B. (2007). *CRC Handbook of Avian Body Masses*. Boca Raton, FL: CRC.

[BIO060370C48] Ellington, C. P. (1984). The aerodynamics of hovering insect flight. I. The quasi-steady analysis. Philosophical Transactions of the Royal Society of London. B, Biological Sciences, 305, 1-15.

[BIO060370C19] Fernández, M. J., Springthorpe, D. and Hedrick, T. L. (2012). Neuromuscular and biomechanical compensation for wing asymmetry in insect hovering flight. *J. Exp. Biol.* 215, 3631-3638. 10.1242/jeb.073627 22771747

[BIO060370C20] Feinsinger, P. and Chaplin, S. B. (1975). On the relationship between wing disc loading and foraging strategy in hummingbirds. *Am. Nat.* 109, 217-224. 10.1086/282988

[BIO060370C21] Goodall, C. (1991). Procrustes methods in the statistical analysis of shape. *J. R. Stat. Soc. Series B Stat. Methodol.* 53, 285-339. 10.1111/j.2517-6161.1991.tb01825.x

[BIO060370C22] Graham, C. H., Supp, S. R., Powers, D. R., Beck, P., Lim, M. C. W., Shankar, A., Cormier, T., Goetz, S. and Wethington, S. M. (2016). Winter conditions influence biological responses of migrating hummingbirds. *Ecosphere* 7, e01470. 10.1002/ecs2.1470

[BIO060370C23] Groom, D. J. E., Toledo, M. C. B., Powers, D. R., Tobalske, B. W. and Welch, K. C. (2018). Integrating morphology and kinematics in the scaling of hummingbird hovering metabolic rate and efficiency. *Proc. R. Soc. B.* 285, 20172011. 10.1098/rspb.2017.2011 PMC5832699 29491168

[BIO060370C24] Gunz, P. and Mitteroecker, P. (2013). Semilandmarks: a method for quantifying curves and surfaces. *Hystrix* 24, 103-109. 10.4404/hystrix-24.1-6292

[BIO060370C25] Hedrick, T. L., Tobalske, B. W., Ros, I. G., Warrick, D. R. and Biewener, A. A. (2012). Morphological and kinematic basis of the hummingbird flight stroke: scaling of flight muscle transmission ratio. *Proc. R. Soc. B.* 279, 1986-1992. 10.1098/rspb.2011.2238 PMC3311889 22171086

[BIO060370C26] Hou, L., Verdirame, M. and Welch, K. C. (2015). Automated tracking of wild hummingbird mass and energetics over multiple time scales using radio frequency identification (RFID) technology. *J. Avian. Biol.* 46, 1-8. 10.1111/jav.00478

[BIO060370C27] Klingenberg, C. P. and Rohwer, S. (2013). Visualizations in geometric morphometrics: how to read and how to make graphs showing shape changes. *Hystrix* 24, 15. 10.4404/hystrix-24.1-7691

[BIO060370C28] Langston, N. E. and Rohwer, S. (1996). Molt-breeding tradeoffs in albatrosses: life history implications for big birds. *Oikos* 76, 498-510. 10.2307/3546343

[BIO060370C29] Lind, J. and Jakobsson, S. (2001). Body building and concurrent mass loss: flight adaptations in tree sparrows. *Proc. R. Soc. B.* 268, 1915-1919. 10.1098/rspb.2001.1740 PMC1088827 11564347

[BIO060370C30] Magnan, A. and Sainte-Laguë, A. (1933). Le vol au pointe fixe. In: *Exposés de morphologie Dynamique et de Mécanique du Mouvement (Actualités Scientifiques et Industrielle #60)* (ed. M. A. Magnan), pp. 1-31. Paris: Hermann et Cie.

[BIO060370C31] Mcguire, J. A., Witt, C. C., Remsen, J. V., Corl, A., Rabosky, D. L., Altshuler, D. L. and Dudley, R. (2014). Molecular phylogenetics and the diversification of hummingbirds. *Curr. Biol.* 24, 910-916. 10.1016/j.cub.2014.03.016 24704078

[BIO060370C32] Mcnab, B. K. (1988). Food habits and the basal rate of metabolism in birds. *Oecologia* 77, 343-349. 10.1007/BF00378040 28311947

[BIO060370C33] Meijering, E., Dzyubachyk, O. and Smal, I. (2012). Methods for cell and particle tracking. *Methods Enzymol.* 504, 183-200. 10.1016/B978-0-12-391857-4.00009-4 22264535

[BIO060370C34] Mitteroecker, P. and Gunz, P. (2009). Advances in geometric morphometrics. *Evol. Biol.* 36, 235-247. 10.1007/s11692-009-9055-x

[BIO060370C35] Osborne, M. F. M. (1951). Aerodynamics of flapping flight with application to insects. *J. Exp. Biol.* 28, 221-245. 10.1242/jeb.28.2.221 14850630

[BIO060370C36] Palmer, R. S. (1972). Patterns of molting. In: *Avian Biology*, 2 (ed. D. S. Farner, J. R. King and K. C. Parkes), pp. 65-101. London: Academic Press, Inc.

[BIO060370C37] Rajabi, H., Dirks, J.-H. and Gorb, S. N. (2020). Insect wing damage: causes, consequences and compensatory mechanisms. *J. Exp. Biol.* 223, jeb215194. 10.1242/jeb.215194 32366698

[BIO060370C38] Rayner, J. M. (1988). Form and function in avian flight. In *Current Ornithology*, 5 (ed. R. F. Johnston), pp. 1-66. Boston, MA: Springer US.

[BIO060370C39] R Core Team. (2022). R: A language and environment for statistical computing. R Foundation for Statistical Computing, Vienna, Austria. https://www.R-project.org/.

[BIO060370C40] Rohlf, F. J. (1999). Shape statistics: procrustes superimpositions and tangent spaces. *J. Classif.* 16, 197-223. 10.1007/s003579900054

[BIO060370C41] Rohlf, F. J. (2021a). tpsDig, digitize landmarks and outlines, version 2.32. Department of Ecology and Evolution, State University of New York at Stony Brook.

[BIO060370C42] Rohlf, F. J. (2021b). tpsSmall, testing amount of shape variation, version 1.36. Department of Ecology and Evolution, State University of New York at Stony Brook.

[BIO060370C43] Russell, S. M., Russell, R. O., Pollock, J. and Hill, A. (2001). *The North American Banders’ Manual for Banding Hummingbirds.* California: North American Banding Council.

[BIO060370C44] Sane, S. P. and Dickinson, M. H. (2002). The aerodynamic effects of wing rotation and a revised quasi-steady model of flapping flight. *J. Exp. Biol.* 205, 1087-1096. 10.1242/jeb.205.8.1087 11919268

[BIO060370C45] Sargent, A. J., Groom, D. J. E. and Rico-Guevara, A. (2021). Locomotion and energetics of divergent foraging strategies in hummingbirds: a review. *Integr. Comp. Biol.* 61, 736-748. 10.1093/icb/icab124 34113992

[BIO060370C46] Schindelin, J., Arganda-Carreras, I., Frise, E., Kaynig, V., Longair, M., Pietzsch, T., Preibisch, S., Rueden, C., Saalfeld, S., Schmid, B. et al. (2012). Fiji: an open-source platform for biological-image analysis. *Nat. Methods* 9, 676-682. 10.1038/nmeth.2019 22743772 PMC3855844

[BIO060370C47] Shankar, A., Graham, C. H., Canepa, J. R., Wethington, S. M. and Powers, D. R. (2019). Hummingbirds budget energy flexibly in response to changing resources. *Funct. Ecol.* 33, 1904-1916. 10.1111/1365-2435.13404

[BIO060370C49] Stiles, F. G. (1995). Intraspecific and interspecific variation in molt patterns of some tropical hummingbirds. *Auk.* 112, 118. 10.2307/4088772

[BIO060370C50] Suarez, R. K. (1992). Hummingbird flight: sustaining the highest mass-specific metabolic rates among vertebrates. *Experientia* 48, 565-570. 10.1007/BF01920240 1612136

[BIO060370C51] Swaddle, J. P. and Witter, M. S. (1997). The effects of molt on the flight performance, body mass, and behavior of European starlings (Sturnus vulgaris): an experimental approach. *Can. J. Zool.* 75, 1135-1146. 10.1139/z97-136

[BIO060370C52] Tobalske, B. W., Warrick, D. R., Clark, C. J., Powers, D. R., Hedrick, T. L., Hyder, G. A. and Biewener, A. A. (2007). Three-dimensional kinematics of hummingbird flight. *J. Exp. Biol.* 210, 2368-2382. 10.1242/jeb.005686 17575042

[BIO060370C53] Tucker, V. A. (1973). Bird metabolism during flight: evaluation of a theory. *J. Exp. Biol.* 58, 689-709. 10.1242/jeb.58.3.689

[BIO060370C54] Vogel, S. (1981). *Life in Moving Fluids: The physical biology of Flow*. Princeton, NJ: Princeton University Press.

[BIO060370C55] Walsberg, G. (1983). Avian ecological energetics. In: *Avian Biology VII* (ed. D. S. Farner, J. R. King and K. C. Parkes), pp. 161-220. London: Academic Press, Inc.

[BIO060370C56] Warrick, D. R., Tobalske, B. W. and Powers, D. R. (2005). Aerodynamics of the hovering hummingbird. *Nature* 435, 1094-1097. 10.1038/nature03647 15973407

[BIO060370C57] Watanabe, A. (2018). How many landmarks are enough to characterize shape and size variation? *PLoS One* 13, e0198341. 10.1371/journal.pone.0198341 29864151 PMC5986137

[BIO060370C58] Weis-Fogh, T. (1972). Energetics of hovering flight in hummingbirds and in Drosophila. *J. Exp. Biol.* 56, 79-104. 10.1242/jeb.56.1.79

[BIO060370C59] Wilcox, S. C. and Clark, C. J. (2022). Sexual selection for flight performance in hummingbirds. *Behav. Ecol.* 33, 1093-1106. 10.1093/beheco/arac075

[BIO060370C60] Zelditch, M. L., Swiderski, D. L. and Sheets, H. D. (2012). *Geometric Morphometrics for Biologists: A Primer*. Amsterdam: Elsevier Academic Press.

[BIO060370C61] Zenzal, T. J., Diehl, R. H. and Moore, F. R. (2014). The impact of radio-tags on Ruby-throated Hummingbirds (*Archilochus colubris*). *Condor* 116, 518-526. 10.1650/CONDOR-13-142.1

