# Supplementary material for: Winging it: hummingbirds alter flying kinematics during molt
Source: Biol Open. 2024 Nov 11;13(11):bio060370. doi: 10.1242/bio.060370 (PMC11583918; doi:10.1242/bio.060370)
Supplement: Supplementary information [file biolopen-13-060370-s1.pdf]

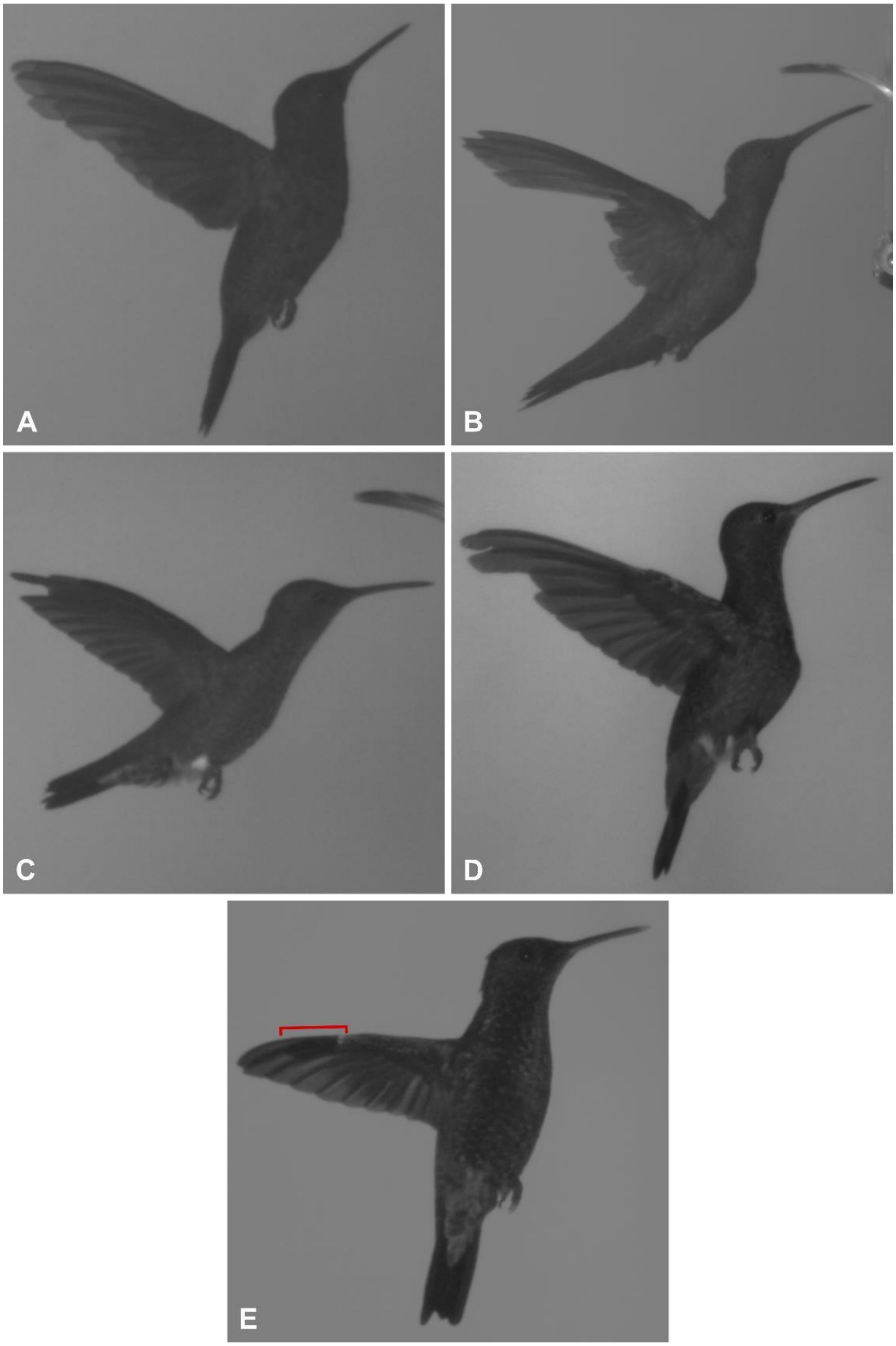

Fig. S1. Molting hummingbirds classified by the degree of molt. (A) Saucerottia cyanifrons individual experiencing low molt degree of inner primaries. (B) *Anthracothorax nigricollis* individual experiencing high molt degree of inner primaries. (C-D) Amazilia tzacatl individuals experiencing low molt degree of outer primaries. (E) *Saucerottia cyanifrons* individual experiencing high molt degree of outer primaries. The red bracket points to a primary feather emerging from its sheath; notice that this feather overlaps with others, therefore it does not contribute to wing area.

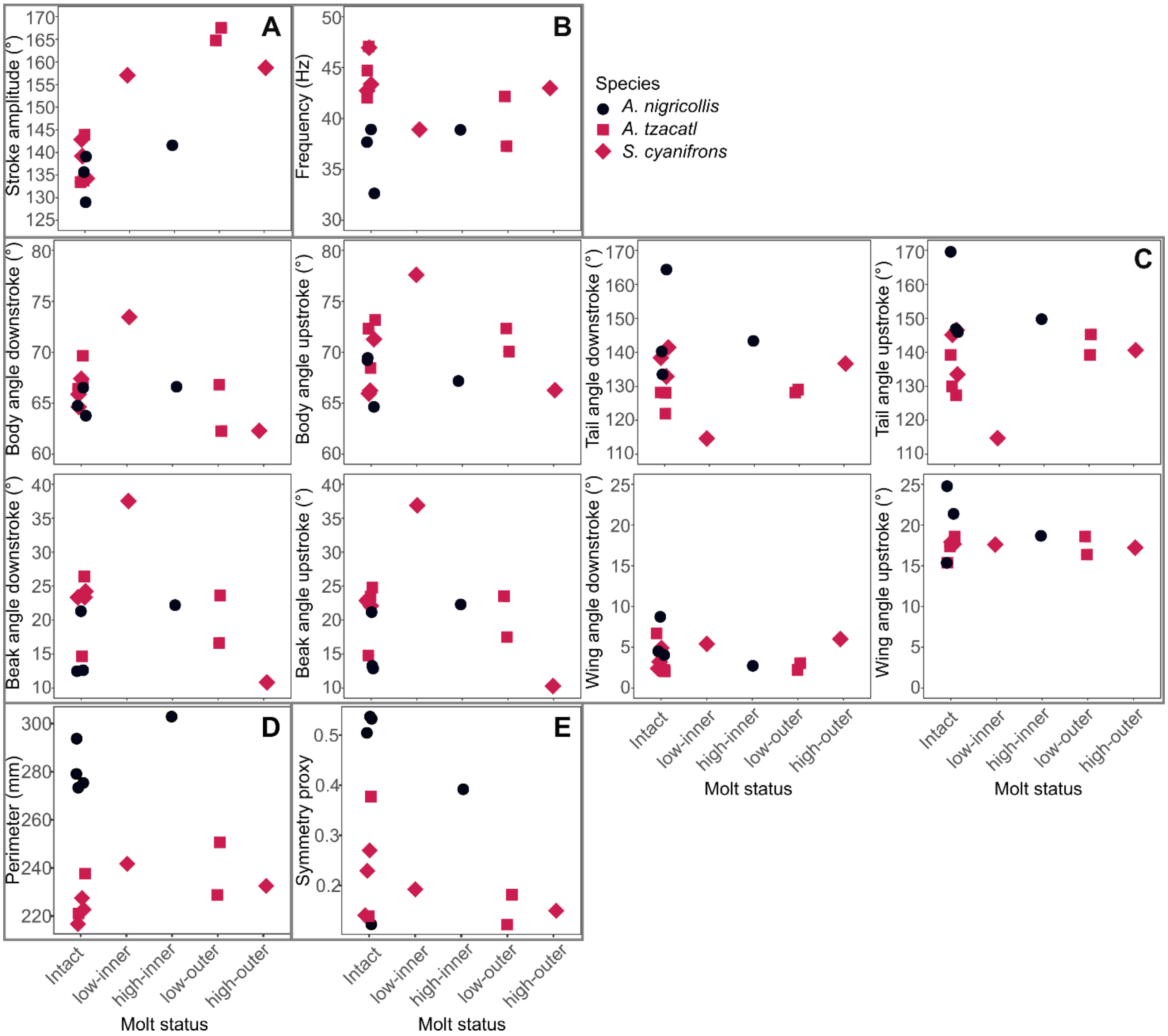

Fig. S2. Kinematic variables per species and degree of molt. There is no clear trend regarding how molting degree (categories in the x axes; Fig. S1) affects the kinematic or morphometric variables. (A) Molting hummingbirds showed higher stroke amplitudes for every species, but the difference was greater in the emeralds (red) compared to the mango (black). (B) Molting and non-molting hummingbirds showed similar wing flapping frequency, yet more variation in flapping frequency ranges was observed in non-molting birds. (C) Side-view angles did not appear to be different between molting and non-molting birds. (D) Perimeter of analemmas. (E) Ratio of anterior loop to analemma length. Colors represent different hummingbird clades: mangos (black) and emeralds (red).

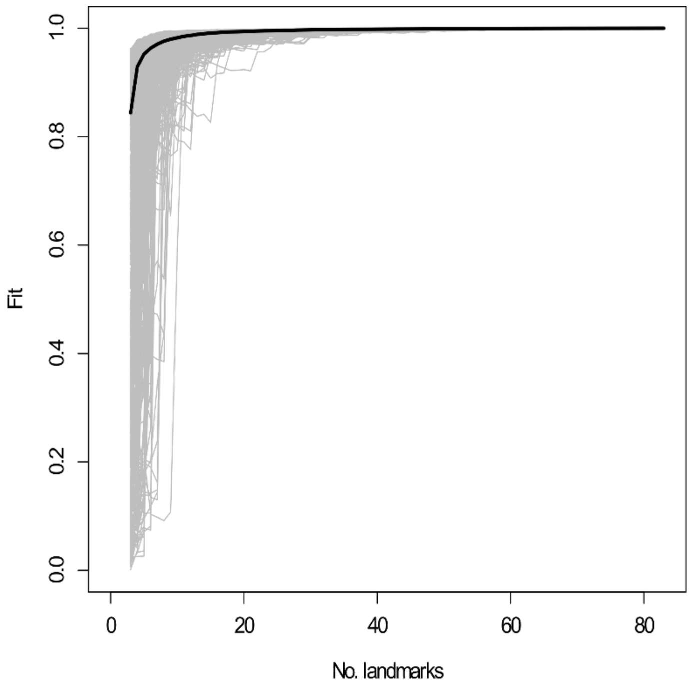

Fig. S3. Sampling curve from performing LaSEC to estimate the sufficient number of landmarks and semilandmarks to characterize analemma shape variation. LaSEC (Watanabe, 2018) performs a GPA using the full set of landmarks and semilandmarks to obtain the distribution of specimens in the shape space, then selects random subsamples varying the number of landmarks and semilandmarks, and computes the fit between the subsample specimen distribution and that of the full dataset measured as Procrustes sum of squares. Gray lines indicate fit values from different iterations of subsampling (iterations=10000). The dark line represents the median fit value at each number of landmarks.

Note that around 20 landmarks a plateau in the characterization of shape variation is reached. Based on this analysis, we decided to work with 39 landmarks and semilandmarks.

Biology Open • Supplementary information

Table S1. GLM results for the top view flight kinematics variables. Molting hummingbirds exhibit differences in flight kinematics compared to non-molting hummingbirds. The models did not find species or the interaction (molt-species) effect on flight kinematics variables (downstroke, upstroke, and stroke amplitude).

Dependent variable:			
	Downstroke	Upstroke	Stroke amplitude
	(1)	(2)	(3)
Molt	-8.930***	-13.115**	19.107***
	(2.225)	(5.664)	(4.157)
Species (*S. cyanifrons* vs *A. nigricollis*)	2.193	-1.003	-4.180
	(1.990)	(5.066)	(3.718)
Species (*S. cyanifrons* vs *A. tzacatl*)	1.610	-0.107	-1.739
	(1.990)	(5.066)	(3.718)
Interaction (Molt: *S. cyanifrons* vs *A. nigricollis*)	5.037	6.528	-12.150
	(3.588)	(9.133)	(6.703)
Interaction (Molt: *S. cyanifrons* vs *A. tzacatl*)	-5.440	-5.098	10.016
	(3.147)	(8.010)	(5.879)
Constant	18.800***	29.320***	138.790***
	(1.407)	(3.582)	(2.629)
Observations	14	14	14
Akaike Inf. Crit.	70.843	97.004	88.345
Note:		*p<0.1; **p<0.05; ***p<0.01	

Table S2. Number of filmed individuals and number of analemmas analyzed by species and molting state. In the non-molting individuals column, the numbers before the semicolon represent the individuals used for angle and frequency analyses, and the numbers after the semicolon, the individuals used for geometric morphometric analysis.

Species	Molting individuals	Non-molting individuals	Analemmas of molting individuals	Analemmas of non-molting individuals
Anthracothorax nigricollis	1	3;4	3	35
Amazilia tzacatl	2	3;2	13	8
Saucerottia cyanifrons	2	3;3	14	12
Total	5	9	30	55

Biology Open • Supplementary information

Table S3. GLM results for top view flight kinematics variables, comparison made among clades: Mangos (*A. nigricollis*) and Emeralds (*S. cyanifrons* and *A. tzacatl*). Molt has a significant effect on all flight kinematics variables. In addition, the model results suggest that molt effects vary among clades, as seen in the interaction between molt and clade.

Dependent variable:			
	Downstroke	Upstroke	Stroke amplitude
	(1)	(2)	(3)
Molt	-11.650***	-15.664***	24.115***
	(1.659)	(3.737)	(3.156)
Clade (Mangos vs Emeralds)	1.388	-0.950	-3.310
	(1.818)	(4.093)	(3.457)
Interaction (Molt: Mangos vs Emeralds)	7.757**	9.077	-17.158**
	(3.401)	(7.658)	(6.467)
Constant	19.605***	29.267***	137.921***
	(1.050)	(2.363)	(1.996)
Observations	14	14	14
Akaike Inf. Crit.	71.458	94.185	89.452
Note:		*p<0.1; **p<0.05; ***p<0.01	

Biology Open • Supplementary information
